# 3D Carbon Nanostructures Derived from 2D Irida-Graphene: Insights into Structural, Mechanical, Electronic, and Optical Properties

**DOI:** 10.1021/acsomega.5c05013

**Published:** 2025-08-20

**Authors:** Isaac M. Felix, Raphael B. de Oliveira, Luiz A. Ribeiro, Douglas S. Galvão, Marcelo L. Pereira, Raphael M. Tromer

**Affiliations:** † Center for Agri-food Science and Technology, 154624 Federal University of Campina Grande, Pombal, Paraíba 58840-000, Brazil; ‡ Department of Applied Physics and Center for Computational Engineering and Sciences, State University of Campinas, Campinas, São Paulo 13083-970, Brazil; § Department of Materials Science and NanoEngineering, 3990 Rice University, Houston, Texas 77005, United States; ∥ Institute of Physics, 28127 University of Brasília, Brasília, Federal District 70910-900, Brazil; ⊥ College of Technology, Department of Electrical Engineering, 28127 University of Brasília, Brasília, Federal District 70910-900, Brazil

## Abstract

The exploration of three-dimensional (3D) carbon allotropes has received increasing attention due to their potential in advanced materials and nanotechnology. Irida-Graphene (IG), a two-dimensional carbon allotrope with a structure consisting of 3–6–8 carbon rings, can be used as a precursor for creating 3D materials with tailored properties. This work presents a comprehensive computational characterization of four novel 3D structures derived from IG, named 3D-IG-α, -β, -γ, and -δ. These structures were generated through biaxial strain and layer compression, followed by detailed analyses of their structural, electronic, mechanical, and optical properties. Stability was confirmed via density functional theory optimizations and ab initio molecular dynamics simulations at 800 K, demonstrating structural integrity under high-temperature conditions. Electronic analyses revealed indirect band gaps ranging from 0.62 to 1.68 eV, indicating semiconducting behavior. Mechanical analyses revealed anisotropic Young’s modulus values. Optical properties exhibit strong absorption and reflectivity in the ultraviolet range, making them potential candidates for UV-blocking materials.

## Graphical abstract

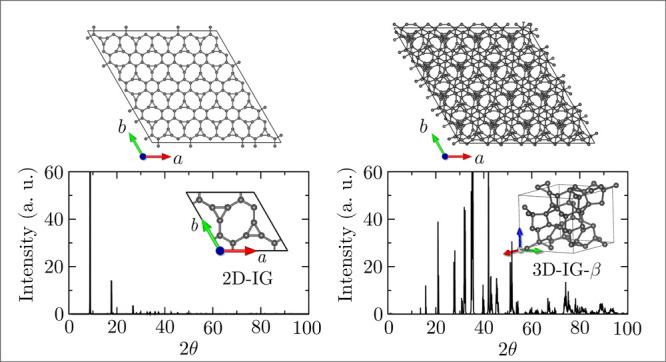

## Introduction

The discovery of new carbon allotropes has become a significant focus in materials science, driven by the potential to uncover unique properties and enable innovative applications across a wide range of technologies.^1^ Among these, graphene^2^ stands out as a groundbreaking material that has revolutionized the field. Carbon’s versatility in forming various topologies and hybridizations enables the design of materials with tunable properties tailored to specific needs.^3–5^

One of the recent developments is Irida-Graphene (IG), a two-dimensional (2D) carbon allotrope composed of 3-, 6-, and 8-membered carbon rings.^6^ IG has garnered growing attention due to its remarkable characteristics: it is metallic, planar, and thermally stable up to 1000 K. Moreover, with a Young’s modulus of approximately 400 GPa, IG demonstrates good mechanical strength.

Recent works have expanded the scope of IG investigations. Studies on chemical functionalization have shown that decorating IG with metallic atoms, such as lithium (Li),^7,8^ sodium (Na),^8–10^ titanium (Ti),^11^ potassium (K),^8^ and calcium (Ca)^12^ can optimize its performance for hydrogen storage, achieving gravimetric densities that exceed the standards set by the US Department of Energy (DOE). Specifically, Li-decorated IG and Ca-decorated IG exhibited reversible hydrogen storage capacities of up to 8.0 wt % with high structural stability and optimal performance under ambient conditions.^7,12^ Furthermore, boron-doped and Na-decorated systems (Na@BIG) demonstrated exceptional storage capacities of 8.8 wt %,^10^ highlighting their promising potential for future technological applications.

In the energy storage domain, IG has also shown promise as an electrode material for lithium-ion batteries (LIBs),^13,14^ sodium-ion batteries (SIBs),^14–16^ potassium-ion batteries (KIBs),^14^ and magnesium-ion batteries (MIBs).^17^ Density Functional Theory (DFT) simulations revealed that IG layers exhibit high electrical conductivity, low ion diffusion barriers (ranging from 0.07 to 0.24 eV), and elevated estimated capacities, reaching up to 1643 mAh/g in MIB systems.^17^ This combination of high energy storage density and mechanical stability positions IG as an effective candidate for green energy storage devices.

Other IG-based allotropes have been proposed, further expanding their properties and applications. Twin Irida-Graphene, composed of 3–16–8 carbon rings, exhibits an anisotropic response to polarized light and a high dielectric constant, making it suitable for optoelectronic applications.^18^ Irida-Graphyne, with sp and sp^2^ hybridizations, also exhibits high optical absorption and structural dynamical stability.^19^ Moreover, variants based on nitrogen, boron, and silicon, such as Irida-B_12_N_12_ (Ir-BN) and Irida-Silicene (ISi), exhibit dynamic, thermal, and electronic stabilities along with optical activity in visible and ultraviolet regions.^20,21^

Nanostructures, such as IG Nanoribbons (IGNRs), have also demonstrated good performance, depending on their edge configurations. Zigzag configurations (ZIGNRs) exhibited metallic behavior with high Seebeck coefficients and excellent thermoelectric properties, while armchair configurations (AIGNRs) demonstrated semiconducting behavior with an electronic band gap of 2.4 eV.^22^ These findings offer valuable insights into optimizing nanomaterials for advanced electronic and thermoelectric applications.

The IG physicochemical properties have also been explored. Studies indicated that IG thermal conductivity is isotropic, approximately 215 W/mK at room temperature, lower than graphene due to phonon scattering.^23^ Mechanical properties, such as fracture stress for monolayer and bilayer structures, were also evaluated, highlighting structural robustness and potential for nanoelectronic devices.^24^ Despite these contributions, research has predominantly focused on the 2D form of IG, leaving the transition to its related three-dimensional (3D) structures still unexplored.

The present work is based on a recently proposed approach to create 3D carbon allotropes from 2D precursors through a systematic topological transformation process.^25^ This approach starts with selecting a stable 2D carbon allotrope, which is then subjected to biaxial in-plane compression (along the *x* and *y* directions) and subsequent compression along the *z*-axis. The in-plane compression induces structural buckling, which enhances the chemical reactivity of the 2D layers, thereby facilitating the formation of covalent bonds among layers during the *z*-axis compression. This stepwise process enables the transformation of a planar 2D system into a 3D one while maintaining control over its geometric and electronic properties.^25^

In the present study, we have applied this methodology to derive four novel corresponding 3D IG configurations (3D-IG), named 3D-IG-α, -β, -γ, and -δ, respectively. The structural stability of these structures was validated through simulations based on density functional theory (DFT) and ab initio molecular dynamics (AIMD) simulations at elevated temperatures. Electronic properties revealed a semiconducting behavior with indirect electronic band gaps ranging from 0.62 to 1.68 eV. Furthermore, significant mechanical anisotropy and strong absorption in the ultraviolet region were observed, highlighting these structures as promising candidates for semiconductor, optoelectronic devices, and protective coating applications.

## Methodology

To generate a 3D structure from 2D IG layers, we developed a computationally optimized protocol applicable to a broad range of carbon allotropes. This procedure begins by arranging three IG layers (totalizing 36 carbon atoms per unit cell) with an initial interplanar spacing of 3 Å. Controlled biaxial stresses were systematically applied along the *xy* plane, then followed by compression along the *z*-axis to induce covalent interlayer bonding. These steps resulted in cohesive and stable 3D-IG configurations. Among the stress levels tested, 2, 4, 6, and 8% were identified as effective in obtaining structurally stable and mechanically robust structures.

Initial structural optimization and geometry evaluations were performed using the Molecular Orbital PACkage (MOPAC2016).^26^ MOPAC was used successfully in the process of obtaining 3D structures from 2D ones, and vice versa.^25^

To further investigate the properties of the obtained 3D configurations, DFT calculations were performed using the Spanish Initiative for Electronic Simulations with Thousands of Atoms (SIESTA) code.^27^ A double-ζ valence (DZV) basis set was employed to accurately represent the wave functions. At the same time, exchange-correlation effects were treated using the generalized gradient approximation (GGA) with the Perdew–Burke–Ernzerhof (PBE) functional.^28,29^ The interactions between valence and core electrons were described using Troullier–Martins norm-conserving pseudopotentials.^30^

A kinetic energy cutoff of 400 Ry was employed to balance computational efficiency and precision. Structural optimizations utilized a 5 × 5 × 5 *k*-point mesh, whereas a denser 15 × 15 × 15 mesh was employed for the electronic structure and projected density of states (PDOS) calculations. Both atomic positions and lattice vectors were fully relaxed during the optimization process. The convergence criterion was set to a maximum residual force of less than 0.05 eV/Å on any atom.

The energetic stability of the proposed 3D structures was initially assessed by calculating their cohesive energy (*E*_coh_), defined as

$$E_{\mathrm{coh}}=\frac{E_{3D-\mathrm{system}}-n\cdot E_{C}}{n}$$ 1

where *E*_3D–system_ is the total energy of the relaxed structure, *E*_C_ is the energy of an isolated carbon atom, and *n* is the number of atoms in the unit cell.

To further evaluate the dynamical stability, phonon dispersion relations were obtained at zero temperature using the finite displacement method, as implemented in the SIESTA package. Displacements of 0.10 Å were applied along the Cartesian directions, and the resulting forces were used to compute the force constants. The same numerical settings and *k*-point grid adopted for the electronic structure calculations were employed to ensure convergence.

Finally, the thermal stability of the systems was examined through AIMD simulations performed at 800 K in the canonical (NVT) ensemble. A time step of 1 fs was used, and the system temperature was regulated via a Nosé-Hoover thermostat over a simulation period of 5 ps, to verify structural integrity under thermal fluctuations.

The mechanical properties of the 3D structures were characterized by determining Young’s modulus values under both tensile and compressive strains. Simulations were carried out at a strain of 1%, within the linear elastic regime along the *x*, *y*, and *z* directions. Young’s modulus values were obtained from the slope of the stress–strain curve in the linear region, providing insights into the stiffness and elastic behavior of the material under different loading conditions.

Optical properties were determined from the complex dielectric function, ϵ­(ω), where the imaginary part, ϵ_2_(ω), was calculated using Fermi’s golden rule,^31^ and the real part, ϵ_1_(ω), was obtained via the Kramers–Kronig relations.^32–34^ Using these components, key optical properties such as the absorption coefficient α, refractive index η, and reflectivity *R* were derived following the expressions outlined in^35,36^

$$\alpha (\omega )=\sqrt{2}\omega [(\epsilon_{1}^{2}(\omega )+\epsilon_{2}^{2}(\omega ){)}^{1/2}-\epsilon_{1}(\omega ){]}^{1/2}$$ 2

$$\eta (\omega )=\frac{1}{\sqrt{2}}[(\epsilon_{1}^{2}(\omega )+\epsilon_{2}^{2}(\omega ){)}^{1/2}+\epsilon_{1}(\omega ){]}^{1/2}$$ 3

and

$$R(\omega )={\left[\frac{(\epsilon_{1}(\omega )+i\epsilon_{2}(\omega ){)}^{1/2}-1}{(\epsilon_{1}(\omega )+i\epsilon_{2}(\omega ){)}^{1/2}+1}\right]}^{2}$$ 4

## Results and Discussion

We have investigated 3D structures derived from 2D IG,^6^ employing a previously proposed methodology for transforming layered 2D systems into 3D crystalline materials.^25^ This approach involves compressing layers of the 2D precursor to induce covalent bonding, allowing the formation of stable 3D crystals.

The 2D IG system was subjected to biaxial strain applied along the monolayer plane before compression. The lattice was then systematically compressed or decreased along the *z*-direction at increments of 0.5 Å, starting from an initial interlayer spacing of 3 Å, forming several 3D structures. Biaxial strain values ranging from 1 to 10% were explored, and the most stable geometries were selected for detailed analysis. As mentioned above, four distinct configurations, corresponding to biaxial strain levels of 2, 4, 6, and 8%, were identified and designated as 3D-IG-α, -β, -γ, and -δ, respectively.

DFT optimizations were performed to determine the energetic stability of each configuration, followed by ab initio molecular dynamics (AIMD) simulations at 800 K. The zero-temperature optimized geometries of the four 3D-IG phases are depicted in Figure 1, where the unit and 2 × 2 × 2 supercells are displayed along the [100], [010], and [001] crystallographic axes. Although all structures stem from the same 2D precursor, they exhibit pronounced structural diversity, as evidenced by strongly anisotropic lattice parameters in the *x*, *y*, and *z* directions and by the distinct fingerprints in their X-ray diffraction (XRD) patterns.

**Figure 1:**
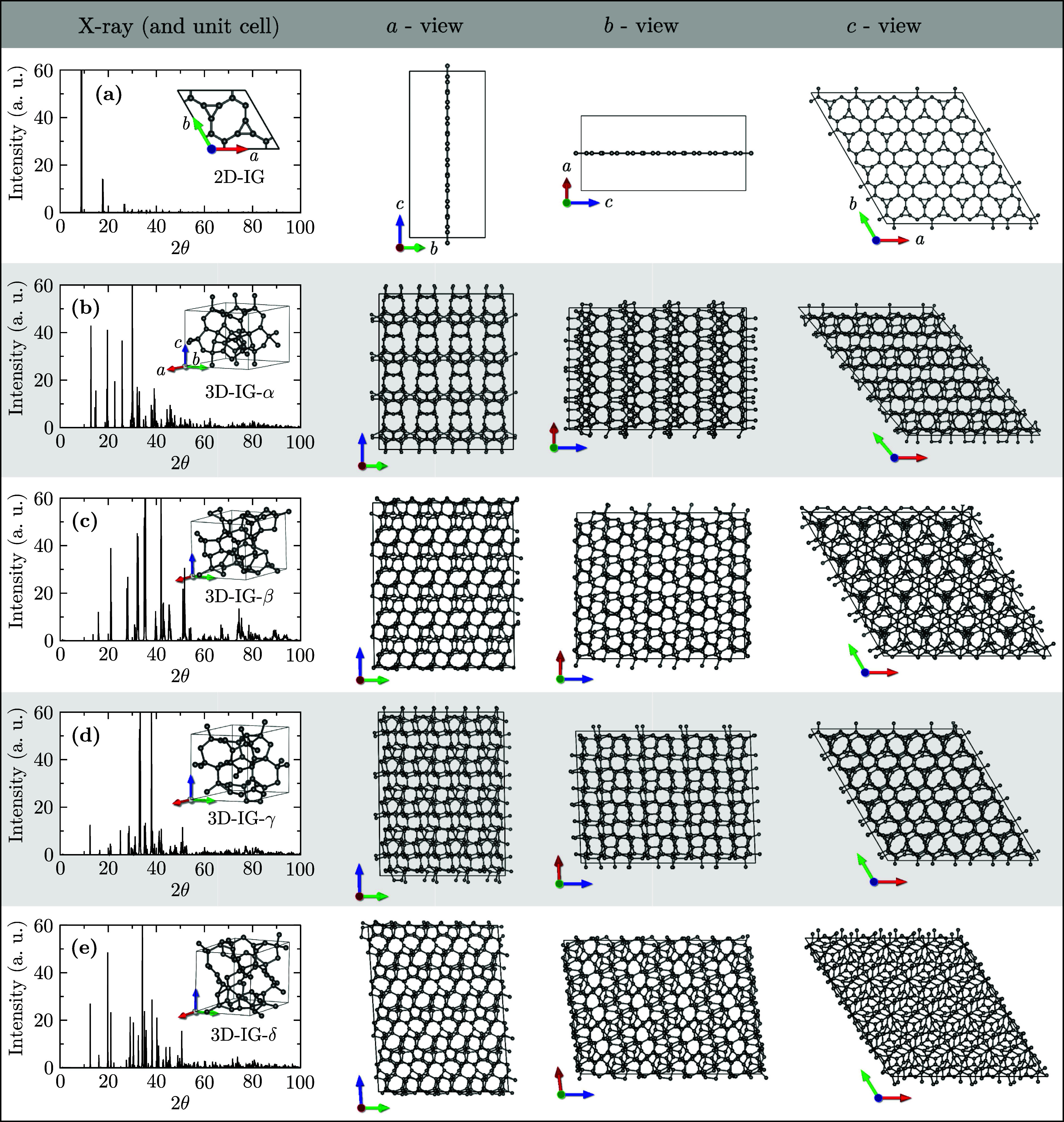
X-ray diffraction patterns and structural representations of 2D IG (a) and its derived 3D-IG-α (b), -β (c), -γ (d), and -δ (e). The leftmost column shows the X-ray diffraction profiles, while the subsequent columns present the unit supercell views along the *a*, *b*, and *c* crystallographic axes.

Table 1 compiles the most relevant crystallographic data: lattice constants (*a*, *b*, *c*), lattice angles (α̂, β̂, γ̂), ranges of C–C bond lengths (Δ*R*_C–C_), bond-angle spreads (Δθ_C–C–C_), cohesive energies (*E*_coh_) and calculated densities (ρ). The bond-length interval extends from 1.32 to 1.63 Å, encompassing the canonical values for both *sp* ^2^ and *sp* ^3^ hybridized carbon. At the same time, the bond-angle distribution spans 102–170°, confirming a broad hybridization spectrum. Among the four phases, 3D-IG-β shows the broadest angular spread (116–157°), indicative of the most complex bonding topology. All configurations adopt the triclinic space group *P*1 (*C*1–1) with no crystallographic symmetry beyond the identity.

**Table 1:** Key Structural Parameters of the Derived 3D-IG-α, -β, -γ, and -δ Configurations^a^

3D-IG	Δ*R* (Å)	Δθ (°)	*a* (Å)	*b* (Å)	*c* (Å)	α̂ (°)	β̂ (°)	γ̂ (°)	*E*_Coh_ (eV/atom)	ρ (g/cm^3^)
α	1.41–1.63	103–133	5.38	6.16	6.90	90.00	90.00	69.32	–8.50	2.80
β	1.50–1.60	116–157	5.53	6.05	6.46	90.14	89.34	112.75	–8.66	3.14
γ	1.33–1.59	104–153	5.44	5.85	7.11	81.90	90.67	113.20	–8.50	2.89
δ	1.32–1.61	102–170	5.60	5.77	7.11	96.38	90.00	114.34	–8.50	2.98

a Table summarizes the range of carbon–carbon bond lengths (Δ*R*_C–C_), bond angle variations (Δθ_C–C–C_), lattice constants (*a*, *b*, *c*), lattice angle deviations (α, β, γ), cohesive energies (*E*_coh_), and densities (ρ).

These metrics contrast with those of the other three-dimensional carbon allotropes. Diamond, formed exclusively by *sp* ^3^ bonds of 1.54 Å, crystallizes in the space group Fd $\overset{-}{3}$ m and has a density of 3.52 g cm^–3^.^37^ Predicted allotropes such as *m*-C_16_,^38^ V-carbon,^39^ Z-carbon,^40^ M-carbon,^41^ and T-carbon^42^ have a wider bond length window (1.49–1.65 Å). By comparison, the densities obtained for the present 3D-IG family (2.80–3.14 g cm^–3^) fall squarely within the interval reported for these alternative 3D carbon phases.^43^

The cohesive energies of 3D-IG-α, -β, -γ, and -δ were estimated as −8.50, −8.66, −8.50, and −8.50 eV/atom, respectively. Among these, 3D-IG-β emerged as the most stable configuration, with a formation energy of approximately 0.16 eV/atom lower than the other structures. This stability correlates with moderate biaxial strain values (e.g., 4%), which optimize the balance between bond lengths and angles, minimizing internal strain within the lattice. In contrast, higher strain levels (e.g., 6 and 8%) lead to decreased stability.

The structural anisotropy observed in the lattice parameters and angles further highlights the diversity among the 3D-IG crystals. For example, 3D-IG-α displays a pronounced deviation in γ̂, reaching 130.24°, indicative of an elongated and distorted lattice. Conversely, 3D-IG-γ and -δ exhibit lattice angles closer to orthorhombic symmetry, with α̂, β̂, and γ̂ values near 90°.

Forming 3D-IG structures through biaxial strain and layer compression allows the creation of multiple stable configurations, each with unique structural and energy properties. Among the four configurations, 3D-IG-β stands out as the most stable, demonstrating optimal hybridization and minimal internal strain. Our study highlights the effectiveness of biaxial strain engineering in tailoring the properties of 3D materials derived from 2D precursors, providing a pathway for designing advanced carbon-based materials with specific functionalities.

Figure 2 presents the phonon dispersion relations for the 3D-IG-α, -β, -γ, and -δ structures, illustrating key vibrational properties and their dynamic stability. The phonon dispersion relations indicate that all systems are dynamically stable, as evidenced by the absence of significant imaginary frequencies across the Brillouin zone. The minimal occurrence of low-magnitude imaginary frequencies near the Γ point suggests that these instabilities are strain-dependent and can be mitigated through small structural adjustments, such as applying minor strain.^44^

**Figure 2:**
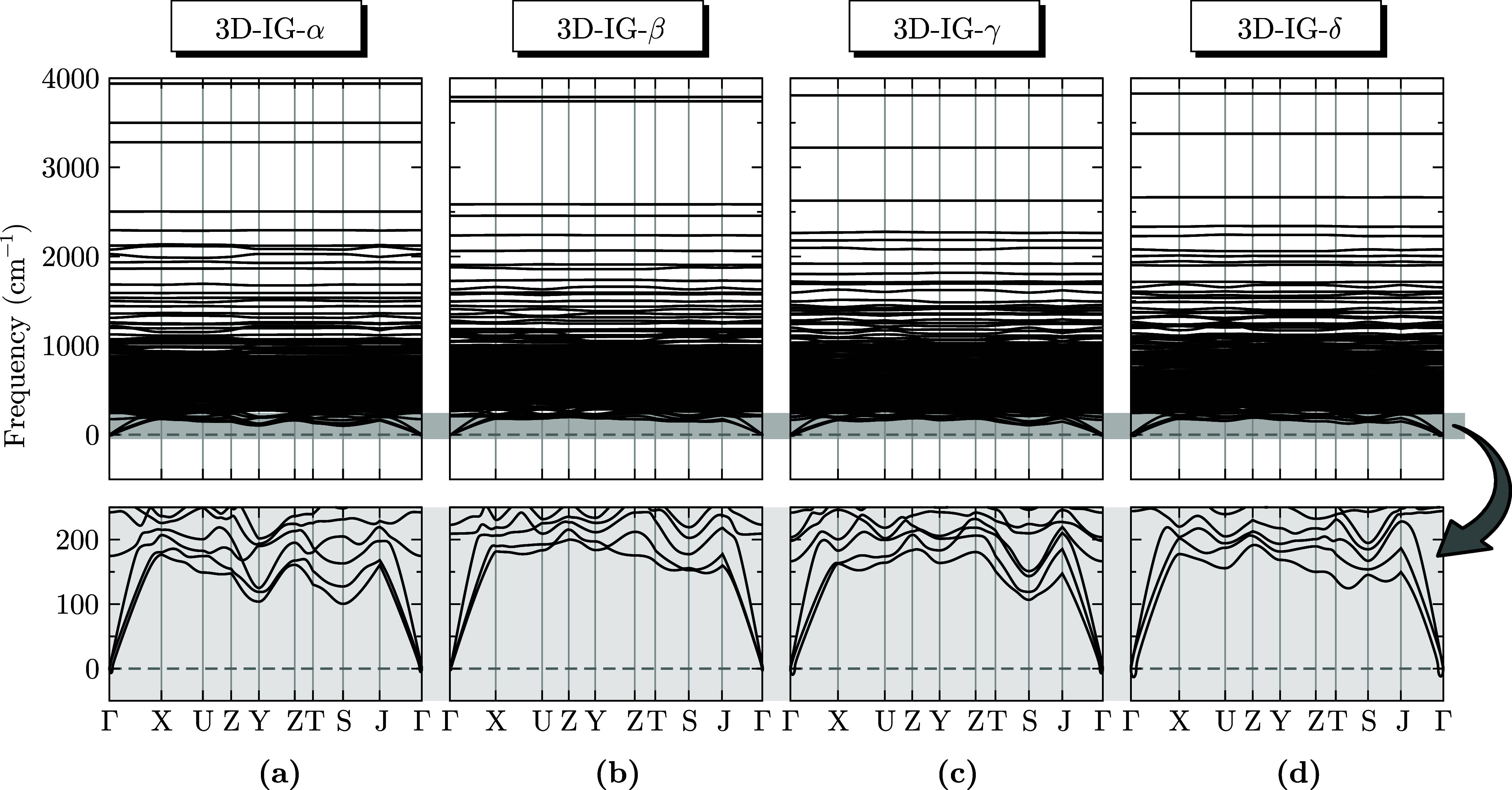
Phonon dispersion relations of the 3D-IG-α (a), -β (b), -γ (c), and -δ (d) structures. The upper panels display the full phonon spectrum, while the lower panels provide a magnified view of the low-frequency range, highlighted in the gray-shaded region.

The high-frequency phonon modes observed in the dispersions are characteristic of strong atomic bonding within the three-dimensional frameworks,^45^ consistent with previous studies on other three-dimensional systems stabilized under high-pressure conditions.^46–48^ While such high-frequency modes are common in high-pressure systems, their observation in 3D carbon-based materials underscores the unique characteristics of the 3D-IG structures. High-frequency phonon dispersions remain an underexplored property in carbon-based systems, offering new insights into their vibrational behaviors.

Building on the confirmed dynamical stability, we conducted AIMD simulations at high temperatures to assess whether these structures retain their structural integrity under thermal stress. Figure 3 presents the final AIMD snapshots of the investigated systems following equilibration at 800 K, along with the corresponding XRD patterns for each structure.

**Figure 3:**
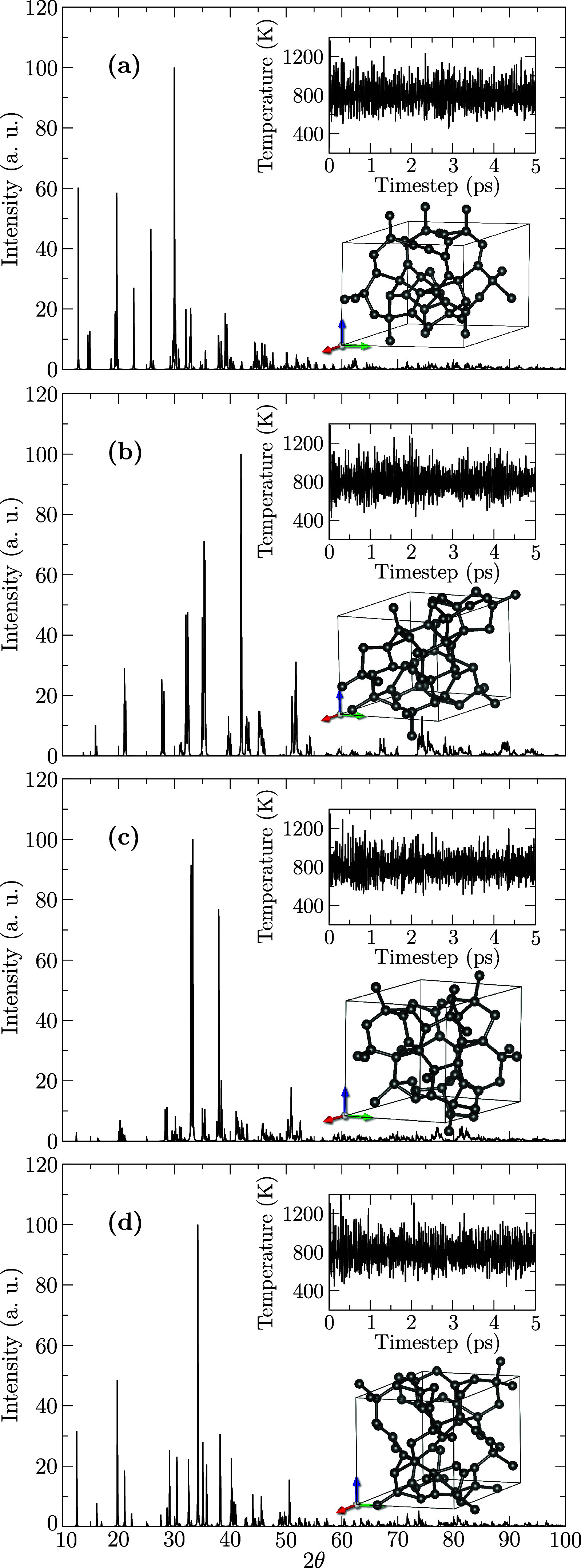
Equilibrium geometries and XRD patterns of the 3D-IG structures obtained from AIMD simulations at *T* = 800 K. Panels (a–d) correspond to the configurations 3D-IG-α, -β, -γ, and -δ, respectively. Insets display the temperature evolution over the simulation time and the final atomic structure of each system within the simulation box.

The results demonstrated that all structures remained intact under thermal stress, exhibiting no evidence of bond breakage or significant structural deformation. A comparative analysis of the XRD patterns revealed a substantial similarity between the high-temperature profiles and the reference patterns shown in Figure 1. This observation provides compelling evidence that the investigated structures preserve their crystalline integrity at elevated temperatures.

We obtained the electronic band structures following rigorous stability tests to evaluate the electronic properties of the 3D structures investigated in this study. These calculations aimed to elucidate the electronic nature of the materials, with the results presented in Figure 4, which depicts both the band structures and the corresponding density of states for the valence bands of the carbon atoms.

**Figure 4:**
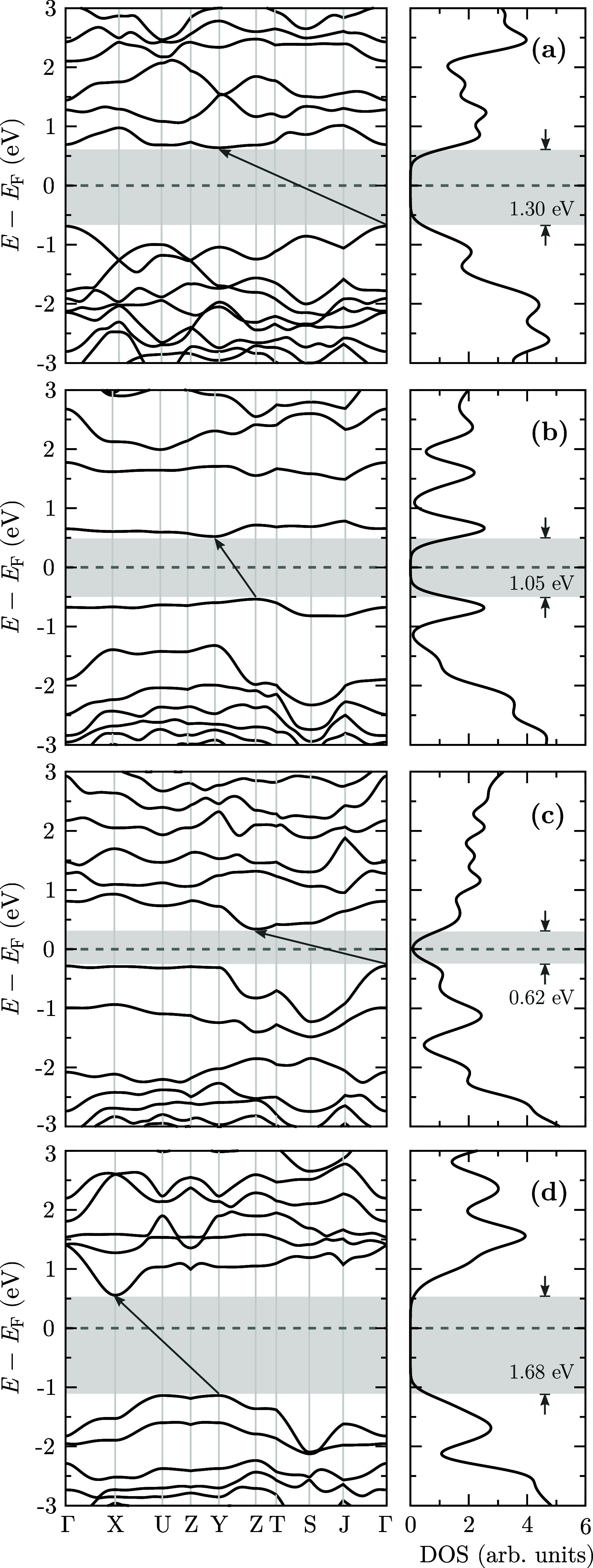
Electronic band structures and the corresponding density of states of the 3D-IG configurations. Panels (a–d) correspond to 3D-IG-α, -β, -γ, and -δ, respectively.

The analysis revealed that all four structures are semiconductors with indirect electronic band gaps. The calculated band gap values are 1.30, 1.05, 0.62, and 1.68 eV for 3D-IG-α, -β, -γ, and -δ, respectively. These indirect transitions occur at distinct points within the Brillouin zone: Γ → Y for 3D-IG-α, Z → Y for 3D-IG-β, Y → Z for 3D-IG-γ, and U → X for 3D-IG-δ. Such variation in transition points reflects the structural diversity among the configurations. The indirect band gap character observed for all investigated 3D-IG structures is a recurrent feature among three-dimensional carbon allotropes. Well-known examples include diamond, M-carbon, V-carbon, and Z-carbon, all of which exhibit indirect band gaps according to prior theoretical studies. At the same level of theory used in this work, employing the GGA-PBE functional, these allotropes present significantly larger band gaps, ranging from 2.26 to 4.48 eV.^37–41^ In contrast, the narrower band gaps calculated for the 3D-IG systems fall within the range typically desired for optoelectronic applications. For instance, in organic photovoltaic devices, semiconductors with band gaps between 1 and 2 eV are favored due to their ability to absorb visible light efficiently and support charge carrier separation.^49^ Finally, it is essential to acknowledge that the PBE functional systematically underestimates band gap values. Nevertheless, it offers a favorable balance between computational cost and qualitative accuracy, as the overall trends it predicts are consistent with those obtained using hybrid functionals.^50^ For instance, in the case of diamond, the discrepancy between PBE and hybrid functional predictions is approximately 23%.^38^

A notable feature observed in the valence band structures of the 3D-IG-β and 3D-IG-γ configurations is the presence of flat bands. Flat band structures are of great interest in condensed matter physics and materials science due to their implications for electronic properties. Flat bands correspond to energy levels nearly independent of momentum, resulting in a high density of electronic states. This phenomenon is particularly intriguing as it may give rise to exotic electronic behaviors, including strong electron correlations and potentially unconventional superconductivity.^51,52^ Future studies could explore whether these structures exhibit emergent phenomena such as ferromagnetism or other strongly correlated effects. However, spin polarization calculations performed for all structures showed no evidence of spin effects, with the total magnetization being zero in all cases.

For the 3D-IG-α, -β, -γ, and -δ structures, charge density maps were generated and are presented in Figure 5, with the charge density regions displayed in red for each configuration. These maps visually represent the spatial distribution of electronic charges within the structures, highlighting areas of high and low localized charge density. This observed distribution is typical of semiconductors.

**Figure 5:**
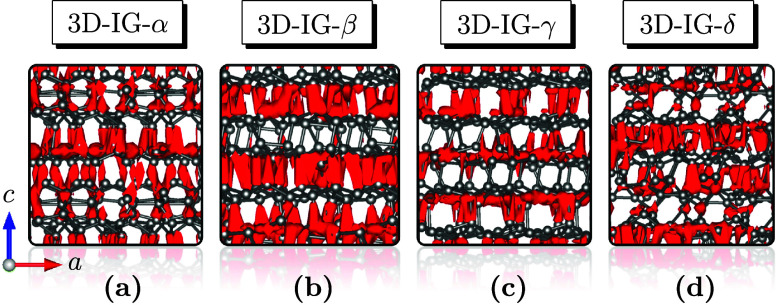
Charge density maps for the 3D-IG-α (a), -β (b), -γ (c), and -δ (d). The red regions represent areas of localized charge density, while the white regions indicate low charge density.

The Young’s modulus values for tensile (*Y*_*M*_^T^) and compressive (*Y*_*M*_^C^) stress along the *x*-, *y*-, and *z*-directions, as summarized in Table 2, provide critical insights into the mechanical behavior and anisotropy of the 3D-IG structures. Compared to diamond, which exhibits an isotropic Young’s modulus of approximately 1100 GPa,^53^ the proposed 3D frameworks display a pronounced directional dependence, with elastic moduli ranging from about 280 GPa to over 780 GPa. For reference, the 2D-IG, formed exclusively by *sp* ^2^ bonds, exhibits a lower modulus of approximately 396 GPa,^6^ falling short of the maximum values reached by its three-dimensional counterparts.

**Table 2:** Young’s Modulus Values for Tensile (*Y*_*M*_^T^) and Compressive (*Y*_*M*_^C^) Stress along the *x*-, *y*-, and *z*-Directions for the 3D-IG-α, -β, -γ, and -δ

3D-IG	(*Y*_*M*_^T^)_*x*_ (GPa)	(*Y*_*M*_^C^)_*x*_ (GPa)	(*Y*_*M*_^T^)_*y*_ (GPa)	(*Y*_*M*_^C^)_*y*_ (GPa)	(*Y*_*M*_^T^)_*z*_ (GPa)	(*Y*_*M*_^C^)_*z*_ (GPa)
α	645.77	683.50	289.48	333.78	584.98	601.42
β	679.54	722.75	748.09	783.75	519.22	528.79
γ	736.15	765.34	747.75	779.11	506.71	464.36
δ	675.74	704.79	580.17	595.29	350.88	380.28

This directional dependence is evident across all 3D-IG configurations. For instance, 3D-IG-α shows its highest stiffness along the *x*-direction (*Y*_*M*_^T^ = 645.8 GPa, *Y*_*M*_^C^ = 683.5 GPa), while presenting reduced moduli along the *z*-axis. Structures such as 3D-IG-β and 3D-IG-γ demonstrate a more balanced mechanical response in the *x*- and *y*-directions, both surpassing the *z*-direction. Notably, 3D-IG-γ exhibits the highest overall stiffness, with tensile moduli of 736.1 and 747.7 GPa along the *x*- and *y*-directions, respectively. In contrast, 3D-IG-δ presents the lowest stiffness, particularly along the *z*-axis (*Y*_*M*_^T^ = 350.9 GPa, *Y*_*M*_^C^ = 380.2 GPa), reinforcing the significant mechanical anisotropy among the studied allotropes.

The complete set of lateral contraction coefficients obtained from the uniaxial tensile and compressive deformations is summarized in Table 3. For each pair of orthogonal axes, the Poisson’s ratio (ν) was evaluated from

$$\nu_{ij}=-\frac{\varepsilon_{j}}{\varepsilon_{i}}$$ 5

where ε_*i*_ is the axial strain applied along the *i*-direction (*i* = *x*, *y*, *z*) and ε_*j*_ is the transverse strain recorded along the *j*-direction (*j* ≠ *i*) under either tensile (T) or compressive (C) loading. Because the present lattices are triclinic, six independent components, ν_*yx*_, ν_*zx*_, ν_*xy*_, ν_*zy*_, ν_*xz*_, and ν_*yz*_, are required to characterize the 3D elastic response.

**Table 3:** Poisson’s Ratios under Tensile ν_*ij*_^*T*^ and Compressive ν_*ij*_^*C*^ Strains for the 3D-IG Structures

3D-IG	ν_*yx*_^*T*^(ν_*yx*_^*C*^)	ν_*zx*_^*T*^(ν_*zx*_^*C*^)	ν_*xy*_^*T*^(ν_*xy*_^*C*^)	ν_*zy*_^*T*^(ν_*zy*_^*C*^)	ν_*xz*_^*T*^(ν_*xz*_^*C*^)	ν_*yz*_^*T*^(ν_*yz*_^*C*^)
α	–0.21 (−0.19)	0.13 (0.14)	0.13 (0.12)	0.11 (0.11)	0.13 (0.13)	0.17 (0.18)
β	–0.10 (−0.10)	0.23 (0.24)	0.19 (0.21)	0.10 (0.12)	0.16 (0.18)	0.08 (0.08)
γ	–0.13 (−0.13)	0.18 (0.17)	0.16 (0.16)	0.12 (0.12)	0.10 (0.14)	0.09 (0.08)
δ	–0.17 (−0.17)	0.22 (0.22)	0.37 (0.34)	0.15 (0.15)	0.12 (0.12)	0.23 (0.22)

Across the four allotropes, the moduli span the interval −0.21 ≤ ν_*ij*_ ≤ 0.37, indicating substantial anisotropy. A distinctive feature is the auxetic character consistently observed for the ν_*yx*_ component. All structures display negative values in the range −0.21 (3D-IG-α) to −0.10 (3D-IG-β). Hence, a uniaxial stretch along *x* leads to a lateral expansion along *y*, a phenomenon that is uncommon but technologically attractive in carbon frameworks.^54^ The remaining components are positive, with the most significant ratios (ν_*xy*_^*T*^ = 0.37 for 3D-IG-δ) still lying below the thermodynamic upper limit of 0.50 for crystalline solids.^55^

A critical value of ν = 0.25 is often adopted to demarcate ductile (ν > 0.25) and brittle (ν < 0.25) regimes.^56^ Although direct transposition to anisotropic systems should be made with caution, the guideline is helpful for a preliminary assessment. Most transverse responses of the 3D-IG family fall below 0.25, suggesting intrinsically brittle behavior, whereas the ν_*xy*_ component of 3D-IG-δ marginally exceeds this threshold, hinting at direction-selective ductility.

To investigate the optical properties of the 3D-IG-α, -β, -γ, and -δ structures, as shown in Figure 6, we analyzed their interaction with polarized electromagnetic radiation over a photon energy range of 0–8 eV. This range encompasses the infrared, visible, and ultraviolet spectra. The electromagnetic radiation was polarized along the *x*-, *y*-, and *z*-directions to capture the directional dependence of the optical response.

**Figure 6:**
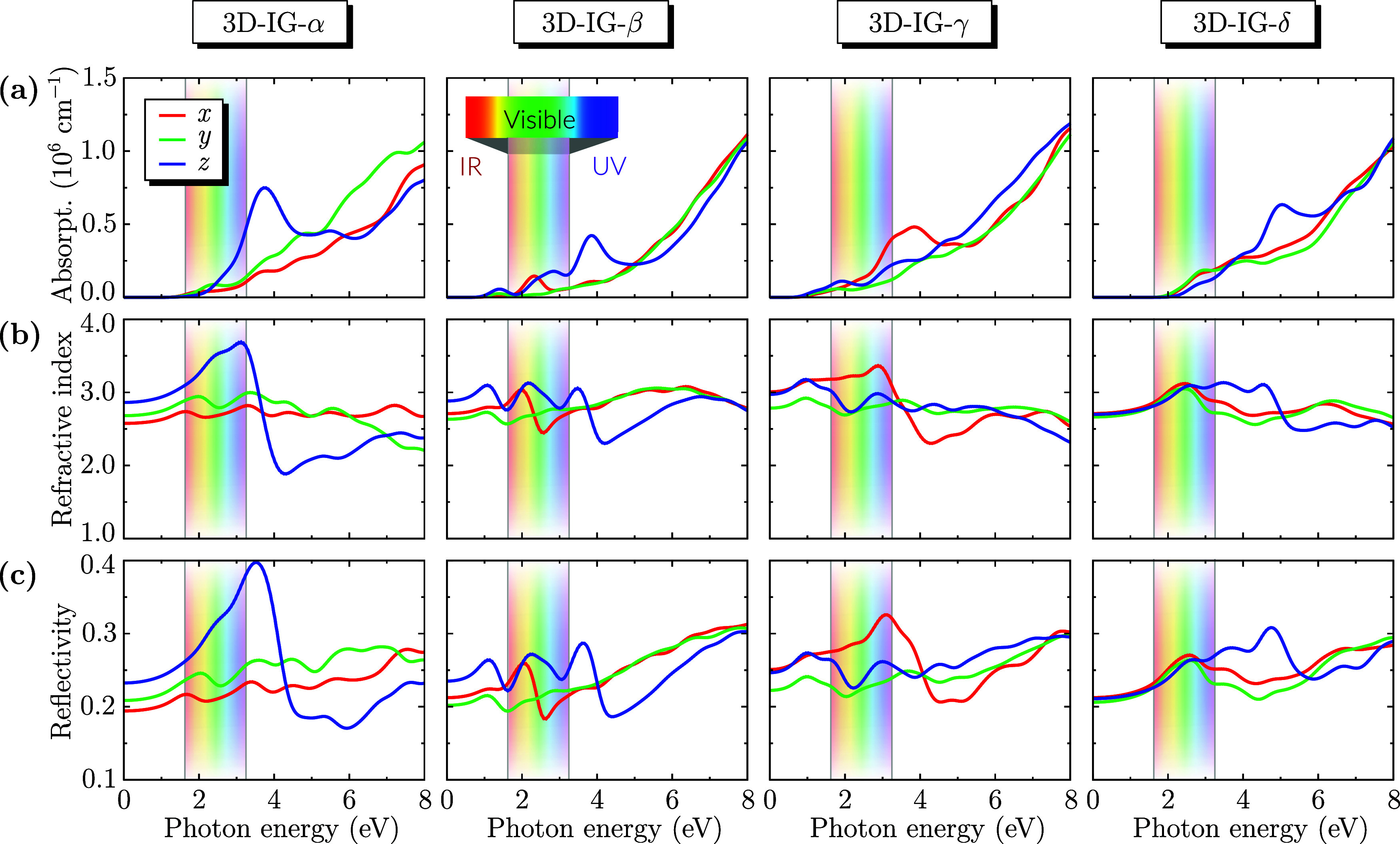
Optical properties of the 3D-IG-α, -β, -γ, and -δ structures as a function of photon energy for electromagnetic radiation polarized along the *x*-, *y*-, and *z*-directions: Absorption coefficient (a), Refractive index (b), and Reflectivity (c). The shaded region indicates the visible light range.

No significant optical activity was observed in the low-energy infrared region. The absorption spectra of the four 3D-IG structures exhibit pronounced anisotropy, with the absorption onset varying significantly depending on the polarization direction. In the case of 3D-IG-α, absorption along the *x*-direction starts around a photon energy of 2.0 eV, while along the *y*-direction, it starts slightly before, and along the *z*-direction, it occurs precisely at 2.5 eV. This directional dependence reflects polarization-specific electronic transitions. Absorption extends into the visible spectrum for all structures.

The refractive index data further corroborate these findings. Near the origin, the curves are nearly flat. They exhibit a plateau for all the systems investigated here and different polarization directions, with exceptions for 3D-IG-α and -γ, which present pronounced peaks around photon energies of 3.0 eV when polarized along the *z*- and *x*-directions, respectively.

Reflectivity as a function of photon energy exhibits similar anisotropy. Starting at approximately 20–30% in the red region, reflectivity gradually increases across the spectrum, reaching nearly 40% in the ultraviolet range. This increase correlates with the decreasing refractive index at higher photon energies, indicating that a significant portion of incident light is reflected at these wavelengths. Consequently, these materials exhibit potential as ultraviolet reflectors, effectively blocking high-energy radiation and protecting against UV exposure.

## Conclusions

This study characterizes novel 3D structures derived from the IG monolayer. By employing biaxial strain and systematic layer compression, we generated four distinct 3D structures, named 3D-IG-α, -β, -γ, and -δ, each exhibiting unique structural, electronic, mechanical, and optical properties. Their structural stability was verified through DFT optimizations and AIMD simulations at 800 K, demonstrating that all structures maintain their structural integrity under high-temperature conditions.

The electronic characterization reveals that all 3D-IG structures are semiconductors with indirect band gaps ranging from 0.62 to 1.68 eV. The valence-band structures of 3D-IG-β and 3D-IG-γ exhibit significant flatness. Spin-polarization analyses confirm the nonmagnetic nature of these materials, with zero total magnetization. Band-gap values in the 1–2 eV window place the 3D-IG phases within the optimal range for visible-light optoelectronics, enabling their integration into all-carbon thin-film photovoltaics, photodetectors, and ambipolar field-effect transistors.

Mechanical-property evaluations demonstrate that Young’s-modulus values of the 3D-IG structures, while lower than those of diamond, remain significant and exhibit pronounced anisotropy. This directional dependence on stiffness, combined with the relatively lower densities of the 3D-IG structures compared to diamond, establishes them as promising candidates for lightweight structural components. The combination of high specific stiffness and low mass density ensures their applicability in microelectromechanical systems (MEMS), nanoscale resonators, and impact-resistant coatings where weight reduction is critical.

Optical analyses further highlight the anisotropic nature of the 3D-IG structures. Strong absorption and reflectivity are observed in the ultraviolet range, with pronounced absorption peaks near 3.5 eV and a monotonic increase extending up to 8 eV, which confirms their suitability for UV-blocking applications. This extended UV response qualifies them for use in transparent UV filters, space-grade protective layers, and photonic architectures requiring direction-selective attenuation of high-energy photons. These findings also point to the feasibility of integrating 3D-IG phases into nano-optoelectronic devices using established thin-film fabrication techniques.
